# Overcoming barriers to cycling for knee disarticulation and transfemoral prosthesis users: A pilot study in The Netherlands

**DOI:** 10.33137/cpoj.v7i2.44191

**Published:** 2024-12-12

**Authors:** F.A de Laat, S.W.M Kühne, W.C.A.J. de Vos, J.H.B. Geertzen

**Affiliations:** 1 Rehabilitation Centre Leijpark, Libra Rehabilitation Medicine & Audiology, Tilburg, The Netherlands.; 2 Livit Ottobock Care, 's Hertogenbosch and Dordrecht, The Netherlands.; 3 University of Groningen, University Medical Centre Groningen, Department of Rehabilitation Medicine, Groningen, The Netherlands.

**Keywords:** Amputation, Cycling, Prosthesis, Mobility, knee Disarticulation, Transfemoral Amputation, Bicycle, Netherlands, Interview, Rehabilitation, Prosthetic Foot

## Abstract

**BACKGROUND:**

Cycling has a number of benefits, especially for individuals with a knee disarticulation or transfemoral prosthesis. However, the barriers they face in cycling are not well understood.

**OBJECTIVES:**

To explore the barriers in cycling experienced by users with a knee disarticulation or transfemoral prosthesis, and to gather solutions to overcome these barriers.

**METHODOLOGY:**

A qualitative research approach was used. In-depth, semi-structured, self-developed interviews were conducted with experienced prosthetic users (N=8) and an adapted version was used for certified prosthetists/orthotists (CPOs) (N=3). The interview included physical, psychological, prosthetic, and bicycle-related items.

**FINDINGS:**

Based on the findings from the interviews, the following barriers and corresponding recommendations were identified:

- Physical barriers: Exertion, skin damage in the groin area and discomfort in the back and hip.
  - Recommendation: Use of an electric bicycle and use of a crank shortener or saddle adjustment to overcome asymmetry in cycling.
- Psychological barriers: Fear of falling or fear of balance disturbances.
  - Recommendation: Task- and context-specific training, or graded exposure to cycling during prosthetic training, along with potentially using a more advanced bike with improved balance.
- Prosthetic barriers: Problems with switching the knee prosthesis mode for cycling; challenges with prosthetic suspension; and discomfort caused by the socket brim design.
  - Recommendation: Manufacturers should integrate automatic detection of cycling in microprocessor prosthetic knee joints; use of a total elastic suspension belt (TES-belt); and lowering the ventral edge of the socket.
- Bicycle-related barriers: Slipping of the prosthetic foot off the pedal.
  - Recommendation: Use of anti-slip pedals or a block heel under the shoe.

**CONCLUSION:**

By addressing the challenges and barriers, we aim to promote greater engagement in cycling, which offers significant physical and psychological benefits for persons with knee disarticulation or transfemoral amputation. Eventually, this can enhance their quality of life and foster greater independence.

## INTRODUCTION

Cycling is an activity with a number of benefits. It can be done as a recreational activity and is a cheap and eco-friendly way of transportation, especially in flat countries like The Netherlands.^1^ For rehabilitation purposes, cycling is a good way to train the cardiovascular system and strengthening the leg muscles.^2^

Persons with a lower limb amputation (LLA) can also benefit from cycling. Cycling is joint friendly, as the majority of body weight is supported by the bicycle seat, thereby reducing the load on the residual limb,^3^ but cycling requires more degrees of flexion at the hip, knee and ankle than walking.^4^ Especially in persons with a LLA due to diabetes, limited joint mobility is common, affecting the range of motion of hip and knee.^5^ To address these limitations in range of motion, adaptations can be made to either the prosthesis or the bicycle.^6^

There are several studies assessing the influence of facilitators and barriers in cycling with a knee disarticulation or transfemoral prosthesis.**7-9** In an older study in Slovenia, persons with a transtibial amputation cycled more than persons with a transfemoral amputation.^7^ In a Thai study, persons with a transtibial amputation were 4.5 times more likely to cycle than persons with a knee disarticulation or transfemoral amputation.^8^ The components of the knee disarticulation and transfemoral prosthesis did not influence the chance in cycling after an LLA, although limited knee flexion ability was mentioned as a barrier. Participants also reported the prosthetic foot slipping off the pedal as a barrier. In that study, almost all participants used their daily prosthesis and shoes while cycling and reported that the prosthetic foot slipping off the pedal was a barrier.^8^ The circumstances in Thailand, however, differ considerably from those in Western countries like The Netherlands, particularly in terms of income and traffic. In a recent study in The Netherlands^9^ an overview was given of facilitators and barriers related to cycling participation in people with an LLA. A dynamic foot positively predicted cycling, whereas adjuvant comorbidity negatively predicted cycling. However, in that study, specific physical and emotional barriers were not mentioned, nor specific factors related to prosthesis or bicycle.^9^

The objective of this pilot study was to explore barriers to cycling and identify solutions by interviewing experienced users of knee disarticulation or transfemoral prostheses, as well as prosthetists working with lower limb amputee cyclists.

## METHODOLOGY

### Participants

Participants were recruited from two orthopedic services in the region of Dordrecht and Eindhoven, The Netherlands. All participants gave informed consent. A waiver from the local Medical Ethical committee was obtained for this study (METC Brabant nr NW2020-41). Inclusion criteria required participants to be regular cyclists with a knee disarticulation or transfemoral amputation. One of the authors (SK) contacted eligible participants, all of whom agreed to participate in the study.

In addition to user opinions, it is important to include the perspectives of certified prosthetists/orthotists (CPOs). Therefore, CPOs with substantial experience (>7 years and working with 10–40 patients who cycle with knee disarticulation or above-the-knee prostheses) were also recruited.

### Procedure

Participants with a knee disarticulation or a transfemoral amputation were asked to participate in an in-depth semi-structured interview with one of the assessors (SK). This interview was self-developed, as no validated interview scheme or questionnaire was available on assessing cycling with a lower limb prosthesis. The interview (in Dutch) comprised open questions about experiences in cycling, barriers in the interaction between prosthesis and bicycle, and recommendations for diminishing these barriers. Characteristics of the participants (age, gender, amputation level, amputation side, type of prosthetic knee and foot, kind of bicycle (motorized or not)) were recorded. The interview framework is provided in **Appendix A**.

For the CPO's, an adapted interview instrument (**Appendix B**) was developed to assess risks associated with cycling using a prosthesis, and barriers related to the interaction between the prosthesis and the bicycle, and possible recommendations and their consequences. The characteristics of the CPOs (years of experience, number of treated persons with an LLA who cycle) were recorded.

### Data analysis

All answers to the interview questions were recorded verbatim, resulting in detailed descriptions of the barriers and recommendations. To ensure the original meaning was preserved, the participants' quotes were translated faithfully in English afterwards by an independent native English-speaking CPO, working in The Netherlands.

These barriers and recommendations were clustered by two independent assessors (FdL and WdV) in 4 categories, based on the global components of the framework of the International Classification of Functioning, Disability and Health (ICF):^10^

**1:** Physical barriers affecting individuals with an LLA, especially skin damage and pain in the back and hips (body functions and structures in the ICF model)
**2:** Psychological barriers affecting individuals with an LLA, especially fear of balance disturbances (personal factors in the ICF model)
**3:** Prosthetic barriers affecting individuals with an LLA, especially the cycle mode in microprocessor knees (MPK's) (environmental factors, [body-related] in the ICF model)
**4:** Bicycle barriers affecting individuals with an LLA, especially the interaction between prosthetic foot and the pedal (environmental factors, [not body-related] in the ICF model).

Differences in clustering between assessors were discussed until consensus was reached.

## RESULTS

### Characteristics of the participants

We approached eight individuals with an LLA (6 with a transfemoral amputation, 2 with a knee disarticulation, numbers 1–8), fulfilling the inclusion criteria, and all agreed to participate. The characteristics of these participants are presented in **Table 1**.

**Table 1:** Participant demographics and prosthetic characteristics.

	Gender (F/M)	Age (y)	Amputation side (R/L)	Amputation level	Type of prosthetic knee	Mechanic (M)/MPK	type of prosthetic foot	Type of prosthetic socket	Liner	Cycling level per week (km)	e-bike (yes/no)
1	F	56	R	TF	C-leg	MPK	Triton	IRC socket	No	0,5–30	no
2	M	52	L	KD	Genium	MPK	Triton HD	Knee disarticulation socket	Yes	0,5–1	no
3	F	71	R	TF	3R106 PRO	M	Trias	Soft brim socket	Yes	0,5–3	yes
4	M	35	R	TF	Rheo Knee	MPK	Variflex Rotate	IRC socket	No	0,5–30	yes
5	M	78	L	TF	VGK	M	Triton	IRC socket	Yes	15	no
6	F	46	L	KD	Genium	MPK	Triton	Knee disarticulation socket (volume adjustable)	Yes	20	no
7	M	66	L	TF	RHEO Knee	MPK	Talux	IRC socket	Yes	25–50	yes
8	F	25	L	TF	Genium	MPK	Trias	Soft brim socket	No	0,5–10	no

Abbreviations: TF, transfemoral; KD, knee disarticulation; MPK, microprocessor knee; IRC socket, transfemoral socket standard with ischial ramus containment (IRC) design.

We also recruited three CPOs (numbers 9–11). They had on average 15 years of experience, and the average number of treated persons with an LLA who cycle was 10–40 per year per CPO.

### Barriers and recommendations in cycling

All barriers, reported more than once, are clustered and presented in **Table 2**.

**Table 2:** Clustered barriers reported by the person with an LLA and their CPO. Abbreviation: CPO, certified prosthetists/orthotists; LLA, lower-limb amputation.

Barriers	Item	Person with an LLA (*n*=8)	CPO (*n*=3)	Total (*n*=11)
		*n*	*n*	*n*
Physical items	Skin damage	6	3	9
	Back pain	4	1	5
	exertion	6	0	6
				
Psychological items	Fear of falling	4	2	6
	Fear of balance disturbances	3	1	4
				
Prosthetic items	Knee prosthesis cycling mode	6	2	8
	Fitting of the socket/suspensi on/socket trim line	5	2	7
				
Bicycle items	Prosthetic foot slipping off the pedal	6	3	9

#### 1) Physical barriers

Skin damage and exertion were the most frequently reported barriers among all the barriers mentioned (82%). Answers of two participants and two CPOs were representative of the physical barriers mentioned:

***1:*** *"Pedaling with just one leg is tiring."*
***5:*** *"Especially the skin that rubs open. In particular in the groin and the top in my hip, where the edges of the prosthesis are located, that presses most on the skin."*
***8:*** *"I had chafing spots with the previous socket, mostly on the hip, because you have more friction there. That socket was quite high and when you make the cycling movement, you continuously get a kind of rubbing effect. The socket connects higher than when walking and, because the cycling motion is greater, it irritated me."*
***9 (CPO):*** *"You can get adhesions in the groin region. These adhesions are between the skin and the socket. While cycling, your movements become viscous, causing a lot of friction, and then the skin eventually breaks… Usually the socket chafes against the skin. In fact, the problems are always in the groin region. Or that they are really starting to get pressure ventrally proximal because the socket is pressing there when cycling."*
***10 (CPO):*** *"Pressure spots … Depends a bit on the position. The socket can start to push in the front and into the groin."*

A recommendation to overcome exertion was to use an electric bicycle. The interaction between the skin and the socket brim during cycling, caused by the constant movement of the thigh, generates shear forces in the groin area. A recommendation was the use of a crank arm shortener, which was unknown for several participants with an LLA. Another recommendation to prevent skin problems is to adjust the saddle or remove the outer part of the saddle on the prosthetic side. In addition, it is recommended to lower the socket and use it in combination with a liner. A socket with soft material on the proximal side (soft-brim socket) is also recommended to reduce problems, if the residual limb is long enough.

Participants reported that back pain was primarily caused by asymmetry during cycling. This asymmetry during cycling has two main causes: First, force application to the pedal is significantly limited with the prosthetic limb, resulting in the majority of the force being generated by the sound limb, which leads to an asymmetrical movement and posture on the saddle. Second, the shape of the socket brim can exert pressure on the groin due to the increased hip flexion required during cycling, which also can lead to an asymmetrical posture on the saddle or cause the upper body to compensate (by leaning backward) in order to avoid discomfort. For this item, the use of a crank arm shortener was proposed again.

#### 2) Psychological barriers

Fear of falling and concerns about balance disturbances were mentioned as psychological barriers. Fear was experienced when stepping on and off the bike, as well as when navigating busy roads. Answers of two participants were representative of the psychological barriers mentioned:

***5:*** *"I have trouble balancing, I am getting older and more anxious… I am afraid I will fall on the prosthetic side."*
***9 (CPO):*** *"Often prosthetic users are afraid of cycling."*

#### 3) Prosthetic barriers

The majority of the participants with an LLA (using a MPK) were dissatisfied with the knee's cycling mode. This is because of the necessity of using a cell phone to activate/deactivate the cycling mode. Some participants with an LLA forgot to switch off the cycling mode after use, which led to dangerous situations.

***1:*** *"It is too cumbersome, it takes too much time. First you have to take your phone, unlock and open the application and connect to the prosthesis, which also takes a while. I think it takes 30 seconds to a minute to activate the bike mode. I do not think it is customer friendly."*
***1:*** *"Apart from that when you get off, you have to take it off bike mode again, otherwise there is a risk of falling."*
***6:*** *"I often forgot to change the knee-setting after cycling."*
***8:*** *"So you have to start it, wait a while … then it can connect. I often have my Bluetooth switched off, because otherwise my battery will drain quickly, so it is frustrating that Bluetooth is still on."*
***10 (CPO):*** *"Attention must be paid to the knee-settings, which have to be changed when getting on and off."*

All participants suggested implementing an automatic detection feature for the cycling mode of the MPK, if not already present. If this is not possible, a second best solution is a switch on the prosthesis (like the Very Good Knee (VGK) knee) instead of using a cell phone.

The fitting of the socket was frequently mentioned as a barrier, due to too much sweating and loosening of the vacuum during cycling.

***8:*** *"When you cycle, there is no vacuum anymore, because air is not anymore pumped out of the socket. If you sweat while doing this, there is also the chance that your socket will slide off your stump. Because you are cycling you do not have the effect that the air is blown or pushed out of the socket."*
***10 (CPO):*** *"The constant rotating movement pushes the socket against the saddle. The socket comes loose from the saddle… which leads to failure of the prosthesis. Constant friction and perspiration can also cause the socket to detach from the stump more quickly."*

**Figure 1:**
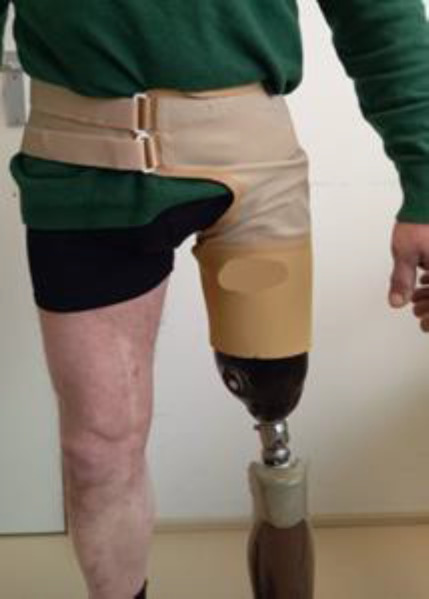
TES-belt. Picture, made by one of the co-authors, with permission.

To overcome fitting problems of the socket, a Total Elastic Suspension (TES) belt was recommended (**Figure 1**).

#### 4) Bicycle barriers

The interaction between prosthetic foot/shoe and the pedal, especially the prosthetic foot slipping off the pedal was most frequently reported.

***2:*** *"I really have to pay attention. Checking whether the foot is properly on the pedal while cycling. Check every time, keep an eye on the foot each time to see if it needs to be repositioned."*
***5:*** *"I adjust the position of my foot and make use of the height of the heel. I then press my heel on the pedal."*
***6:*** *"To prevent the prosthesis from slipping away I had a strap but that is not easy to use. I couldn't get my foot out of it and could not get off my bike in traffic. Also, I had to put the pedal in a straight position before I put my foot in it and then my foot slid in so deep, I could not get it out again. That does not work and is very dangerous."*
***7:*** *"I make sure I put my foot on the pedal correctly, heel behind the pedal, so on good roads nothing dangerous happens."*
***8:*** *"We also bought a click system. But because one had to put shoes on again to cycle, we bought expensive shoes which we never used because I found it too impractical."*
***10 (CPO):*** *"Yes, depending on what type of knee you have. The moment you have a knee that wants to push the knee to extend it slowly moves forward, if it is not locked. Then you have to secure it to the pedal."*

**Figure 2:**
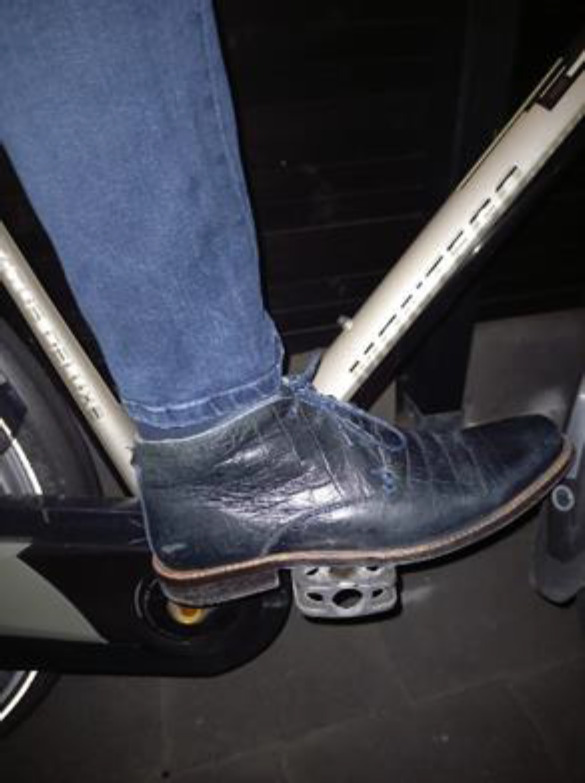
Block heel under the shoe. Picture of one of the co-authors.

To overcome the problem of the prosthetic foot slipping off the pedal, participants with an LLA tried a lot of solutions to click the shoe on the pedal, but frequently unlocking the shoe was dangerous. Two participants recommended a block heel (**Figure 2**) to prevent the prosthetic foot slipping off the pedal and were satisfied with it.

## DISCUSSION

In this study, we assessed barriers to cycling for individuals with a knee disarticulation or transfemoral amputation. We gathered and clustered barriers and recommendations in order to expand the possibilities for cycling. The most commonly mentioned barriers were exertion, residual limb skin damage and the prosthetic foot slipping off the pedal. Additionally, the method of switching the cycling mode on and off in the MPK using a cell phone was mentioned as a problem. Fear of falling, or fear of balance disturbances were the most mentioned psychological items.

In a former Thai study,^8^ the most commonly mentioned barriers were pain and discomfort during cycling. However, in that study, most participants had a transtibial amputation, and none of the participants with a knee disarticulation or transfemoral amputation used an MPK.

To overcome physical problems like skin damage or back pain, a crank arm shortener is proposed. Another option is lowering the edge of the socket, but most of the cyclists use the same prosthesis for cycling as for walking.^8^ Therefore, this solution can disturb the way of walking with the prosthesis and is only possible if the residual limb is long enough. To overcome exertion, an electric bicycle was recommended.

In terms of psychological barriers, fear can be diminished by enlarging self-confidence. The literature suggests that task- and context-specific training, along with graded exposure to cycling, should be initiated as soon as possible to address this issue effectively.^11^ For adults with poor performance and a fear of falling or balance disturbances, the literature suggests that bike adaptations can help, such as a lower frame and automatic saddle height adjustment. These modifications can make it easier to step on and off the bike and ensure that the feet are flat on the ground when standing.^12^

The prosthetic barriers mentioned by the participants were activating and deactivating the cycling mode of the knee and the fitting of the socket. The first item should be addressed by the manufacturers of the microprocessor prosthetic knees by integrating automatic cycling detection. Participants rejected solutions that rely on cell phone operation for mode switching. The fitting of the socket can be improved by using a TES-belt (**Figure 1**). The literature describes an open socket technique for individuals with knee disarticulation, which could be a solution.^13^

The bicycle-related issue that was most frequently mentioned was the prosthetic foot slipping off the pedal. Most of the participants had made adaptations of the pedal, such as larger pedals with anti-slip, or a rubber strip on the lateral side of the pedal, to prevent the shoe from slipping off to the lateral side. These adaptations were satisfactory for the cyclists who made them. Two participants recommended a shoe with a block heel to prevent the prosthetic foot slipping off the pedal (**Figure 2**). Other tried adaptations, such as a toe clip or shoe cleat were not recommended, due to dangerous situations when unlocking.

In summary, individuals with knee disarticulation or transfemoral amputation have the potential to regain their ability to cycle. Prerequisites include a rehabilitation team with the necessary resources, such as an adapted bicycle and/or saddle, expertise in cycle training, and a CPO with experience and interest in cycling, capable of creating an appropriate socket and suspension system.

### Study strengths and limitations

A strength of the study is the broad experience and expertise of the participants, so a lot of barriers and recommendations could be gathered. We included individuals with an LLA ensuring diversity in age, gender, cycling distance levels, and types of prosthetic knees used. It could be seen as a limitation that all participants were recruited in two orthopedic services, so a selection bias could not be excluded. A further limitation of our study was the use of a self-developed interview scheme that was not validated. However, no validated tool existed, nor was there any information from scientific studies to base development of such a tool. This may have had an influence on the outcomes, although the questions asked were as broad as possible, to provide the participants with adequate room for their interpretation. At last it is questionable if saturation occurred in this pilot study. In general, saturation can be achieved in a narrow range (up to 9–17) of interviews, particularly in studies with relatively homogenous study populations and narrowly defined objectives.^14^ During our study, the penultimate participant (a CPO) gave a recommendation to prevent skin damage, whereas the last participant had no new barriers in cycling. Therefore, we think that lack of saturation has at most a minor influence in our results.

## CONCLUSION

There are several barriers in cycling experienced by users with knee disarticulation or transfemoral prosthesis. Addressing these challenges and barriers aims to increase engagement in cycling, thereby providing substantial physical and psychological benefits for this population. Eventually, this can enhance their quality of life and foster greater independence. Future research could focus on multicenter, larger-scale studies with interventions for better cycling, such as an improved socket, or adjustment of the saddle or crank.

## DECLARATION OF CONFLICTING INTERESTS

- Fred de Laat: Nothing to be declared.
- Sabine Kuhne: Employee of Livit Ottobock Care.
- Wouter de Vos: Employee of Livit Ottobock Care.
- Jan Geertzen: Nothing to be declared.

## AUTHORS’ CONTRIBUTION

- **Fred de Laat:** Research design, analysis and interpretation of the data, first draft of the manuscript, manuscript preparation.
- **Sabine Kuhne:** Research design, data-acquisition, analysis and interpretation of the data, manuscript preparation.
- **Wouter de Vos:** Research design, analysis and interpretation of the data, manuscript preparation.
- **Jan Geertzen:** Analysis and interpretation of the data, manuscript preparation.

All authors have read and approved the final version of the manuscript.

## SOURCES OF SUPPORT

None.

## Appendix A Interview Framework for Cycling with a Knee-Disarticulation or Transfemoral Prosthesis.

**Table:**

**Demographics**	Sex: Age: Amputation level: Amputation side: Type of prosthetic knee: Type of prosthetic foot: Type of prosthetic socket: Type of liner: Weekly cycling distance (in kilometers) Type of bike (e-bike or regular bike)	
**Reasons for Cycling**	**Initial Question**	**Follow up Questions**
	Could you tell us the reasons you cycle?	- How often do you cycle? - With whom do you cycle? - How do you feel after cycling? - What kind of feeling do you have after cycling?
**Benefits of cycling**	**Initial Question**	**Follow up Questions**
	In what ways does cycling benefit you?	- Impact on physical health? - Impact on mental health? - Impact on social life? - Impact on independence? - New life possibilities from cycling?
**Barriers to Cycling**	**Initial Question**	**Follow up Questions**
	How was your experience cycling for the first time?	- Why did you want to cycle? - What type of bicycle did you use? - How did it feel to get on and off the bicycle? - How did you manage switching the mode of the MPK knee between cycling and walking? - Was the saddle height comfortable? - Did you feel symmetry in movement between your left and right sides?
	How would you describe your cycling experience?	- Long distance - Short distance - With stopovers
	What barriers do you experience while cycling?	- Socket discomfort - Stump issues, sweating - Prosthetic knee limitations - Prosthetic foot limitations - Movement restrictions (e.g., difficulty bending or extending the hip) - Muscle strength - Pain - Maximum cycling distance - Getting on and off the bike
	Have you ever experienced a dangerous situation in traffic?	- Have you ever fallen off the bike?
**Modifications to the bike**	**Initial Question**	**Follow up Questions**
	Do you have any modifications to your bike?	Typical characteristics of the bike: Backpedal brake Gears Frame height Saddle Click system Adaptations to the bike: Prosthetic or shoe adaptations
	What do you do to make cycling easier?	
	What do you think of a solution to make cycling easier?	

## Appendix B CPOs' Insights on Cycling with Knee-Disarticulation or Transfemoral Prostheses.

**Table:**

Demographics	- Years of work experience: - Number of patients you have treated who cycle:
What bike-related barriers do individuals with lower-limb prostheses face when cycling?	- Stepping on or off the bike - Balance and stability - Type or model of the bike - Other challenges or barriers?
What prosthesis-related barriers do individuals with lower-limb prostheses face when cycling?	- Fit of the prosthesis - Prosthesis length - Socket model - Foot stiffness - Other challenges or barriers?
What solutions have you implemented to address these issues?	- Adaptation: Problem: - Adaptation: Problem: - Adaptation: Problem:
What risks or dangers are associated with cycling using a lower-limb prosthesis?"	- Traffic-related risks - Risks involving other people - Risks related to the bike
